# Overexpression of *HOXA9* upregulates NF-κB signaling to promote human hematopoiesis and alter the hematopoietic differentiation potentials

**DOI:** 10.1186/s13619-020-00066-0

**Published:** 2021-01-11

**Authors:** Jiahui Zeng, Danying Yi, Wencui Sun, Yuanlin Liu, Jing Chang, Lijiao Zhu, Yonggang Zhang, Xu Pan, Yong Dong, Ya Zhou, Mowen Lai, Guohui Bian, Qiongxiu Zhou, Jiaxin Liu, Bo Chen, Feng Ma

**Affiliations:** 1 Chinese Academy of Medical Sciences & Peking Union Medical College (CAMS & PUMC), Institute of Blood Transfusion, No. 26, Huacai Road, Longtan Industry Park, Chenghua District, Chengdu, 610052 China; 2 State Key Laboratory of Biotherapy, Sichuan University, Chengdu, 610065 China; 3 State Key Laboratory of Experimental Hematology, CAMS & PUMC, Tianjin, 300020 China

**Keywords:** Hematopoiesis, Differentiation of hematopoietic lineages, *HOXA9*, Mesoderm, Human embryonic stem cells (hESCs), Tet-on, Inducible expression system

## Abstract

**Background:**

The *HOX* genes are master regulators of embryogenesis that are also involved in hematopoiesis. *HOXA9* belongs to a cluster of *HOX* genes that play extensively studied roles in hematopoiesis and leukemogenesis.

**Methods:**

We established *HOXA9*-inducible human embryonic stem cells (*HOXA9*/hESCs) with normal pluripotency and potential for hematopoiesis, which could be used to analyze gene function with high accuracy. *HOXA9*/hESCs co-cultured with aorta–gonad–mesonephros-derived stromal cells (AGM-S3) were induced to overexpress *HOXA9* with doxycycline (DOX) at various times after hematopoiesis started and then subjected to flow cytometry.

**Results:**

Induction of *HOXA9* from Day 4 (D4) or later notably promoted hematopoiesis and also increased the production of CD34+ cells and derived populations. The potential for myelogenesis was significantly elevated while the potential for erythrogenesis was significantly reduced. At D14, a significant promotion of S phase was observed in green fluorescent protein positive (GFP+) cells overexpressing *HOXA9*. NF-κB signaling was also up-regulated at D14 following induction of *HOXA9* on D4. All of these effects could be counteracted by addition of an NF-κB inhibitor or siRNA against *NFKB1* along with DOX.

**Conclusions:**

Overexpression of *HOXA9* starting at D4 or later during hematopoiesis significantly promoted hematopoiesis and the production of myeloid progenitors while reduced the production of erythroid progenitors, indicating that *HOXA9* plays a key role in hematopoiesis and differentiation of hematopoietic lineages.

**Supplementary Information:**

The online version contains supplementary material available at 10.1186/s13619-020-00066-0.

## Background

The Class I homeobox (*HOX*) genes, which encode a family of homeodomain-containing transcription factors, are master regulators of trunk and tail development during embryogenesis that were initially characterized in *Drosophila (*Sukumar and Shah 2010*)*. In mammals, 39 genes have arisen from the original *HOX* gene cluster through several rounds of duplication, resulting in the formation of four clusters (A, B, C, and D) (Amores et al. 1998).These clusters, which exhibit high levels of conservation and redundancy of function (He et al. 2011), are distributed on human chromosomes as follows: *HOXA*, 7p15; *HOXB*, 17q21; *HOXC*, 12q13; and *HOXD*, 2q31 (Rice and Licht 2007).

*HOXA9*, a homeodomain transcription factor that plays a key role in hematopoiesis, is the gene that is most differentially expressed between CD34+ cells derived from human cord blood (hCB) and human embryonic stem cells (hESCs) ; in particular, the gene is much more strongly expressed in the former lineage (Wang et al. 2005; Domingo-Reines et al. 2017). *HOXA9* is highly expressed in human hematopoiesis stem/progenitor cells (HSPCs) and then down-regulated during differentiation (Sauvageau et al. 1994; Zeng et al. 2019). *Hoxa9-*knockout mice do not have a lethal phenotype, but have a much lower hematopoiesis efficiency (Lawrence et al. 1997). Deficiencies in *Hoxa9* gene lead to myeloid and lymphoid defects and abnormalities in HSC function (Lawrence et al. 1997). *Hoxa9*-/- HSCs cannot rebuild the blood system (Lawrence et al. 2005); conversely, enforced expression of *Hoxa9* in bone marrow HSPCs improves the production of human hematopoietic stem cells (hHSCs) and myeloid precursors (Thorsteinsdottir et al. 2002), and *HOXA9* overexpression in hESC improves hematopoiesis in vitro (Ramos-Mejia et al. 2014). These studies indicate that *HOXA9* plays key roles in hematopoiesis. Overexpression of *HOXA9* in murine marrow cells leads to expansion of HSCs and committed progenitors that likely undergo transformation to acute myeloid leukemia (AML) with co-expression of *Meis1* and *Pbx3*, emphasizing the gene’s key role in blood physiology and pathology (Dassé et al. 2012)

In order to further elucidate the functions of key genes in hematopoiesis, including *HOXA9* , our group developed the Tet-on system based on *piggyBac* with a green fluorescent protein (GFP) tag, PB-Tet-on-OE, and successfully used it to establish transgenic hESC lines (Chen et al. 2017; Zhou et al. 2019). The result is a reliable gene delivery system that can manipulate and track gene expression during early hematopoiesis originating from hESCs with much higher resolution and lower background interference than traditional lentiviral systems (Chen et al. 2017; Zhou et al. 2019; Sun et al. 2019). The aorta–gonad–mesonephros-derived stromal cell (AGM-S3) co-culture system is based on AGM-S3 cells line derived from the stromal cells of AGM region of mouse embryo, which could induce hESC to produce the HSPCs (hematopoietic stem/progenitor cells) and many types of blood cells in vitro (Ma et al. 2008a; Ma et al. 2008b; Ma et al. 2007) , which is a suitable in vitro system for observing the function of *HOXA9* during hematopoiesis. Our results provide clear evidence that overexpression of *HOXA9* promotes hematopoiesis with elevated myeloid potential and weakened erythroid potential, and suggest a molecular/cellular mechanism for its role in hematopoiesis.

## Results

### Transgenic hESCs exhibit inducible expression and normal pluripotency

**Fig. 1:**
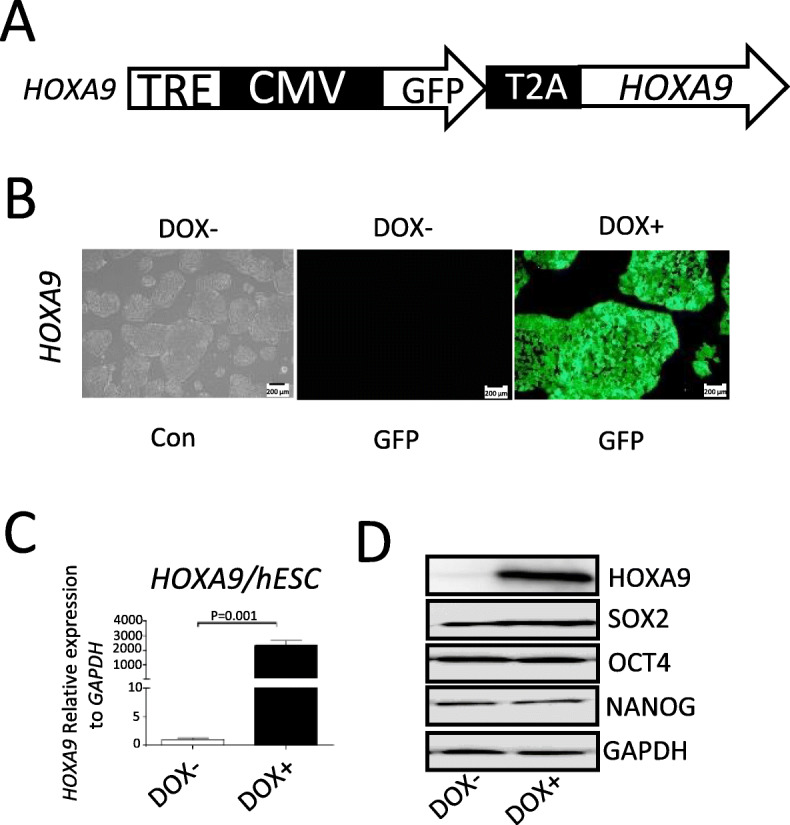
Establishment of inducible *HOXA9* transgenic hESC lines. **a** Schematic representation of the *piggy*Bac constructs used to express *HOXA9*. TRE, tet-on regulation element; CMV mini, cytomegalovirus minimum promoter; T2A, *Thosea asigna* virus 2A peptide. **b** After *HOXA9*/hESCs were induced with DOX for 48 h, the cells were imaged by fluorescence microscopy, allowing observation of co-expressed GFP. **c** qRT-PCR was used to confirm that inducible expression of *HOXA9* was highly stringent and efficient at the transcriptional level. **d** WB detection was used to confirm *HOXA9* overexpression at the protein level, and the pluripotency of *HOXA9*/hESC using ant-SOX2, OCT4, and NANOG antibodies

We constructed PB-Tet-on-GFP-T2A-h*HOXA9* and established the corresponding H1-derived inducible *HOXA9*/hESC line (Fig. 1a). *HOXA9*/hESCs treated with or without DOX for 48 h were monitored by fluorescence microscopy (Fig. 1b), as well as by an qRT-PCR and western blotting (Fig. 1c–d), which revealed highly stringent and efficient induction of *HOXA9*. Western blot analysis showed that stemness-specific markers, including OCT4, SOX2, and NANOG, were expressed at normal levels in *HOXA9*/hESCs (Fig. 1d), demonstrating that these cells have a normal pluripotency potential.

### *HOXA9* overexpression from D4 or later promotes hESC-derived hematopoiesis

**Fig. 2:**
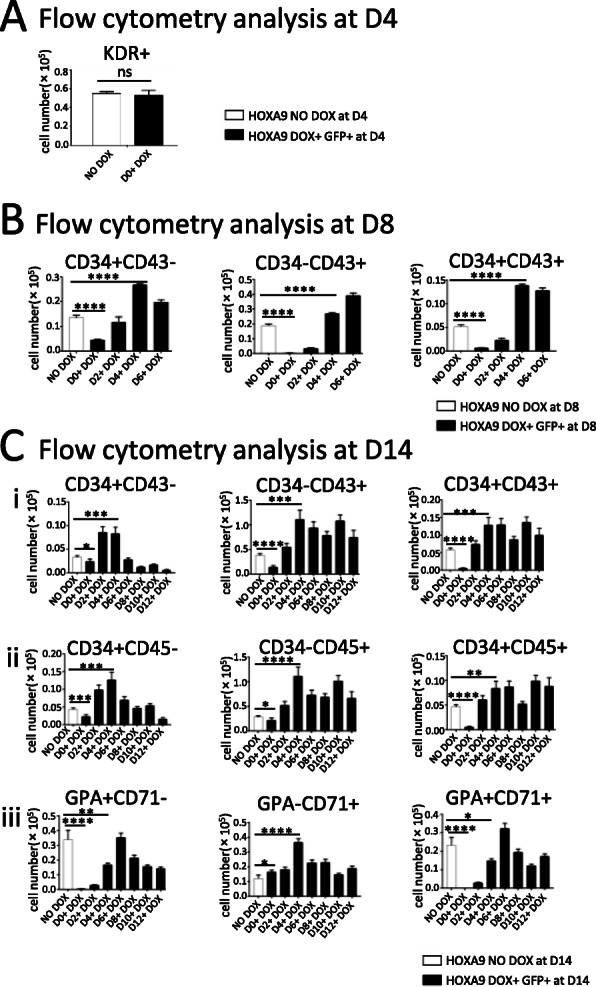
Overexpression of *HOXA9* from D4 promotes hematopoiesis in co-culture with AGM-S3 cells. *HOXA9*/hESCs were treated with DOX starting from D0, D2, D4, D6, D8, D10, or D12 during co-culture with AGM-S3 cells until D14, and the co-culture cells were subjected to flow cytometry analysis using 7-AAD and the following combinations of antibodies: **a** KDR (at D4), **b** CD34/CD43 (at D8), and **c** GPA/CD71, CD34/CD43, or CD34/CD45 (at D14). Results were compared between non-induced co-cultures and the GFP+ fraction of co-cultures treated with DOX starting on different days. When *HOXA9* was induced from the earliest stage, especially D0, CD34 + CD43−, CD34 − CD43+, CD34 + CD43+ populations at D8, and the most hematopoietic populations at D14 were significantly decreased. On the contrary, most above populations at D8 and D14 were dramatically increased by induction of *HOXA9* from D4, especially the CD34 − CD45+ population (but GPA + CD71− and GPA + CD71+ populations were decreased in the most case), indicating that *HOXA9* strongly promotes hematopoiesis except for the early stage. The experiments were repeated three times, and *P* < 0.05 was considered significant (*p < 0.05, **p < 0.01, ***p < 0.001, ****p < 0.0001)

Making use of the Tet-on inducible system, we induced *HOXA9* overexpression during the various stages of hematopoiesis in the AGM-S3 co-culture system and then performed flow cytometry detection at D4, D8, and D14, which results were represented in Fig. 2. When *HOXA9* was overexpressed starting from D0 to D14, the KDR+ cells were not influenced (Fig. 2a, Fig. S2A) while the most hematopoiesis-related populations at D8 and D14 were significantly reduced (Fig. 2b, c, Fig. S2B, S2C), indicating that overexpression of *HOXA9* from the earliest stage did not hinder the induction of mesoderm, but severely blocked human hematopoiesis. But when *HOXA9* was overexpressed from D4 or later it promoted the production of CD34 + CD43−, CD34 + CD43+, and CD34 − CD43+ populations at D8 (Fig. 2b and Fig. S2B), and of CD34 + CD43+, CD34 − CD43+, CD34 + CD45+, especially CD34 − CD45+ populations at D14 except that GPA + CD71+, GPA + CD71− populations at D14 reduced in the most case (Fig. 2c and Fig. S2C). If overexpressed starting from D2 or D4 to D14, the production of CD34 + CD43− and CD34 + CD45− populations was also elevated at D14. These observations indicated that hematopoiesis was significantly enhanced by overexpression of *HOXA9* starting at D4 except for erythrogenesis.

### *HOXA9* overexpression has different effects on hematopoietic potential at different stages of mesoderm development

**Fig. 3:**
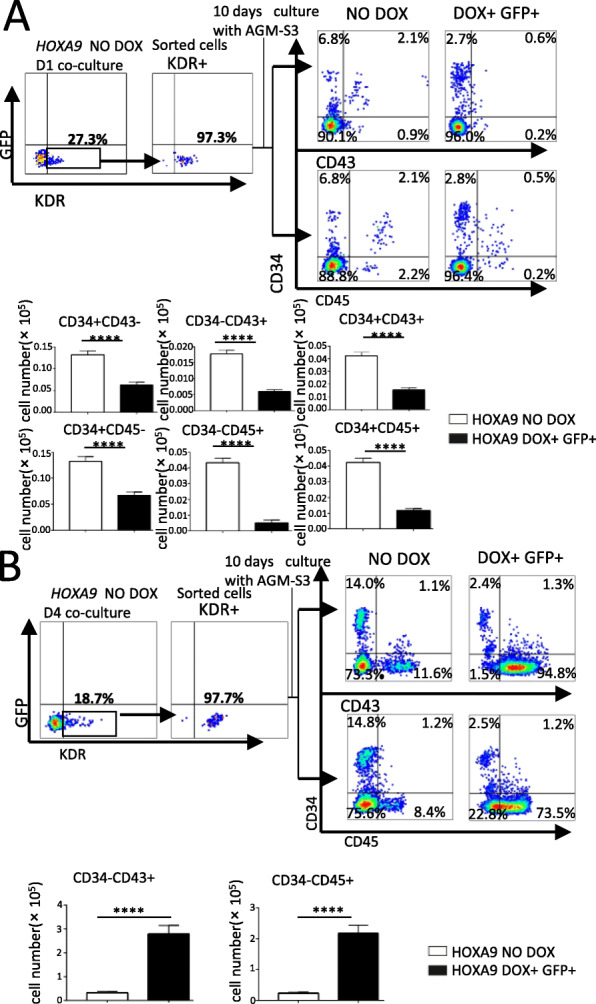
Different effects of *HOXA9* overexpression on the hematopoietic potential of the mesoderm population at different stages. KDR+ cells were sorted from non-induced *HOXA9*/hESC co-cultured with AGM-S3 at D1 (**a**) or D4 (**b**). About 5 × 10^3^ sorted cells were re-plated on irradiated AGM-S3 in 24-well plates, treated with or without DOX, and flow cytometry analysis was performed after 10 days using anti-CD34/CD43/CD45 antibodies. The results revealed that the production of CD34+ cells and their subsequent population could be significantly decreased by overexpression of *HOXA9* from D0 and continued until D14, whereas their production was significantly increased when overexpression of *HOXA9* began on D4 and continued until D14. The experiments were repeated three times, and *P* < 0.05 was considered significant (*p < 0.05, **p < 0.01, ***p < 0.001, ****p < 0.0001)

To elucidate the function of *HOXA9* at different stages of mesoderm development, the key populations with hematopoietic potential, e.g., the KDR+ populations at D1 or D4, were isolated from the corresponding co-cultures, and cultured in the AGM-S3 co-culture system. Overexpression of *HOXA9* blocked the production of CD34+ cells originating from KDR+ cells isolated from co-cultures at D1 (Fig. 3a), whereas for KDR+ cells isolated from co-cultures at D4, *HOXA9* overexpression dramatically increased the production of hematopoiesis-related populations, including CD34 + CD43−, CD34 + CD43+, and CD34 − CD43+, and even CD34 + CD45−, CD34 + CD45+, and CD34-CD45+ (Fig. 3b).

### *HOXA9* overexpression promoted the hematopoietic population at different stages

**Fig. 4:**
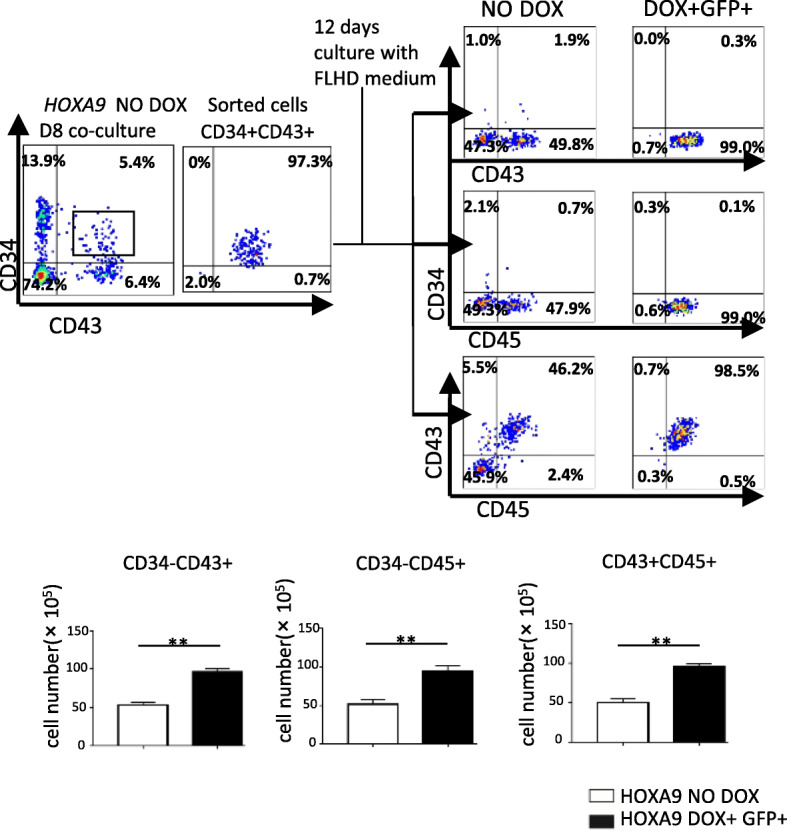
The promotion effects of *HOXA9* overexpressed on CD34 + CD43+ cells at D8. CD34 + CD43+ cells were sorted from the non-induced *HOXA9*/hESCs co-cultured with AGM-S3 at D8. About 5 × 10^3^ sorted cells were re-plated in full-lineage hematopoietic differentiation medium (48-well), treated with or without DOX, and flow cytometry analysis was performed after 12 days using 7-AAD and either combination of anti-CD34/CD43, anti-CD34/CD45 or anti-CD43/CD45 antibodies. All these assays revealed a significant increase in the CD34 − CD43+, CD34 − CD45+, and CD43 + CD45+ populations when *HOXA9* was overexpressed during further culture. The experiments were repeated three times, and *P* < 0.05 was considered significant (*p < 0.05, **p < 0.01, ***p < 0.001, ****p < 0.0001)

**Fig. 5:**
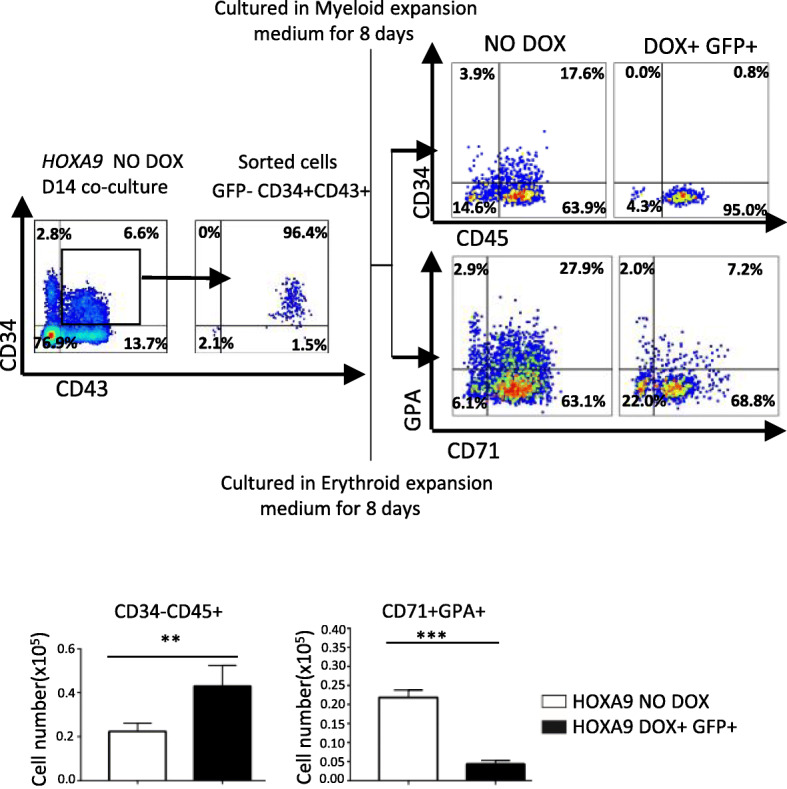
The positive effects of *HOXA9* overexpression on CD34 + CD43+ cells at D14. CD34 + CD43+ cells were sorted from the non-induced *HOXA9*/hESCs co-cultured with AGM-S3 cells at D14. About 5 × 10^3^ sorted cells were resuspended in 250 μl myeloid or erythroid expansion medium and seeded into each well of a 48-well plate, and then treated with or without DOX. flow cytometry analysis was performed after 8 days using 7-AAD and combination of anti-CD34/CD45 or anti-GPA/CD71 antibodies. All of these assays revealed a significant increase in the CD34 − CD45+ population meanwhile decrease in GPA + CD71+ population when *HOXA9* was overexpressed during further culture. The experiments were repeated three times, and *P* < 0.05 was considered significant (*p < 0.05, **p < 0.01, ***p < 0.001, ****p < 0.0001)

To elucidate the function of *HOXA9* on different stages of hematopoiesis, CD34 + CD43+ populations at D8 or D14 were isolated from the corresponding co-cultures and subjected to further culture. The CD34 + CD43+ population sorted from the D8 co-culture were resuspended in full-lineage hematopoietic differentiation medium. Overexpression of *HOXA9* starting on the first day of further culture significantly promoted production of CD45+ cells (Fig. 4). The CD34 + CD43+ population sorted from D14 co-culture was plated in myeloid or erythroid expansion medium. Overexpression of *HOXA9* significantly promoted production of CD45+ cells, but decreased the production of GPA + CD71+ and GPA + CD71− cells (Fig. 5), indicating that *HOXA9* overexpression could markedly promote hematopoiesis, especially myelogenesis, but blocked erythrogenesis.

### *HOXA9* promotes myelogenesis, but impairs erythrogenesis

**Fig. 6:**
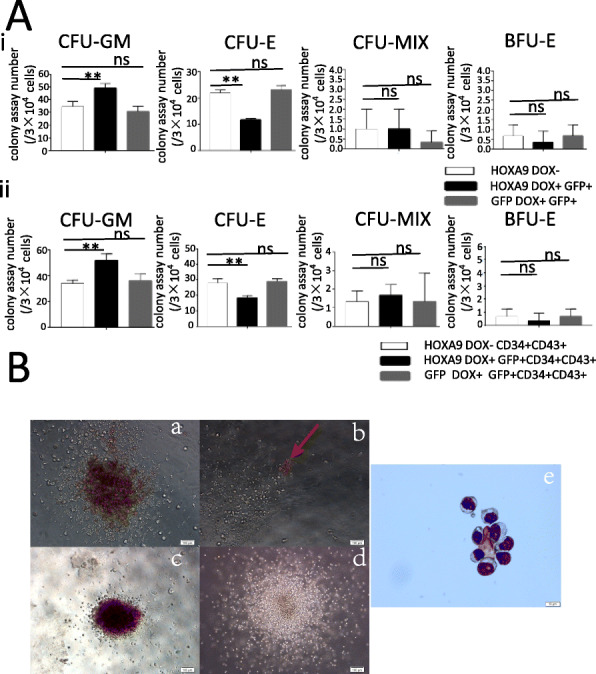
Overexpression of *HOXA9* from D4 increases the myelogenic potential of co-cultured cells while decrease their erythrogenic potential. At D14, the non-induced *HOXA9*/hESC co-culture cells and the GFP+ fractions of D4-induced *HOXA9*/hESC or GFP/hESC co-culture cells, or CD34 + CD43+ cells sorted from these fractions, were subjected to colony culture assays to detect their hematopoietic potentials. (**A**) Percentage of each type of colony derived from 2 × 10^4^ co-cultured cells. (**B**) Typical morphologies of CFU-Mix (a), CFU-E (b), BFU-E (c), and CFU-GM (d) colonies. Scale bar, 100 μm. MGG staining of cells in BFU-E colonies (e). Scale bar, 10 μm. The number of CFU-GM colonies with myelogenic potential increased significantly, but the number of CFU-E colonies with erythrogenic potential decreased, which were not caused by overexpression of GFP. The experiments were repeated three times, and *P* < 0.05 was considered significant (*p < 0.05, **p < 0.01, ***p < 0.001, ****p < 0.0001)

The non-induced *HOXA9*/hESC co-culture cells and the GFP+ cell fractions of *HOXA9*/hESC or GFP/hESC co-culture cells induced from D4, were cultured for colony formation. The number of CFU-E of cells derived from GFP+ cells was less than that of CFU-E of cells derived from non-induced cells, whereas the number of CFU-GM derived from GFP+ cells was greater than that of CFU-GM derived from non-induced cells (Fig. 6Ai). Similar results were obtained for the CD34 + CD43+ population in GFP+ or non-induced cells (Fig. 6Aii). The GFP+ cells or GFP + CD34 + CD43+ cells sorted from D4-induced GFP/hESC co-cultures at D14 obtained similar colony numbers to the corresponding cells sorted from non-induced *HOXA9*/hESC co-cultures. These observations indicate that overexpression of *HOXA9* from D4 can promote the production of myeloid progenitor cells while decreasing the production of erythroid progenitor cells, which effects were not caused by the overexpression of GFP. The morphologies of hematopoietic colonies were confirmed by phase contrast microscopy (Fig. 6B a–d), and erythroid cells were confirmed by May-Grunwald-Giemsa staining (MGG) (Fig. 6B e).

### *HOXA9* overexpression up-regulates NF-κB signaling, and promotion of hematopoiesis by *HOXA9* is abolished by inhibition of NF-κB signaling

**Fig. 7:**
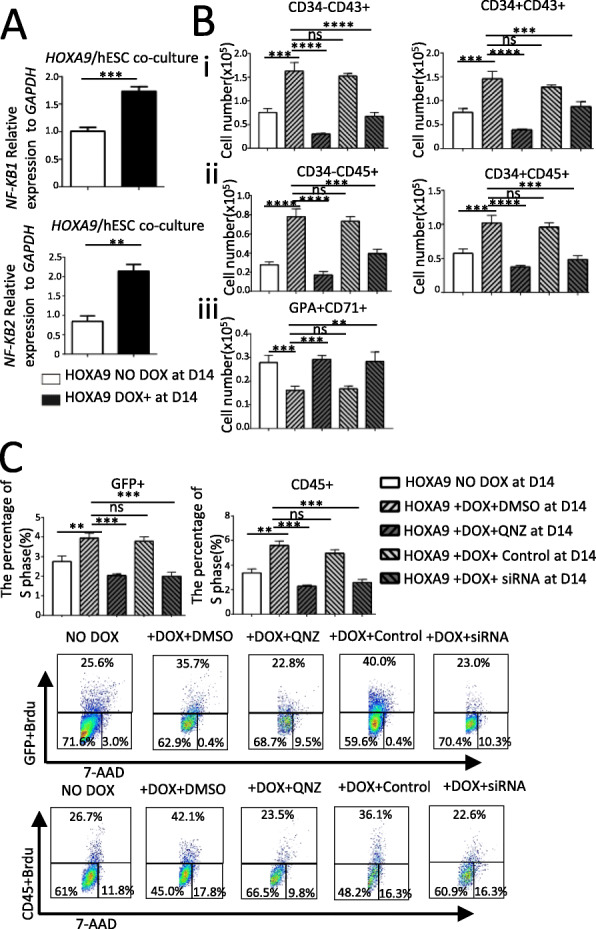
The effects of *HOXA9* overexpression from D4 on hematopoiesis are closely related to NF-κB signaling and might be caused by a change in the cell cycle state. **a** qRT-PCR detection at D14 revealed that expression levels of *NFKB1* and *NFKB2* were significantly enhanced in D4-induced *HOXA9*/hESCs co-cultured with AGM-S3 cells, which indicated that at the late stage NF-κB signaling was up-regulated by *HOXA9* overexpression. **b** D4-induced *HOXA9*/hESCs co-cultured with AGM-S3 cells were treated with 10 nM QNZ (an NF-κB signaling pathway inhibitor) or 20 nM siRNA against *NFKB1*, and flow cytometry analysis at D14 revealed that QNZ or siRNA against *NFKB1* attenuated the positive effects of D4-induced *HOXA9*/hESCs on CD34 − CD43+, CD34 + CD43+, CD34 − CD45+, CD34 + CD45+ populations and the inhibitory effects on CD71 + GPA+ population. **c** Analyses of cell cycle state revealed that *HOXA9* induction from D4 increased the proportion of cells in S phase in co-cultured cells or CD45+ cells expressing *HOXA9* (the GFP+ fraction); this effect was eliminated by treatment with QNZ or siRNA against *NFKB1*. The experiments were repeated three times, and *P* < 0.05 was considered significant (*p < 0.05, **p < 0.01, ***p < 0.001, ****p < 0.0001)

Co-cultures of *HOXA9*/hESCs with AGM-S3 were induced starting at D4, and qRT-PCR detection at D14 revealed that the expression level of the *NFKB1* and *NFKB2* genes was significantly enhanced when *HOXA9* was induced, which indicated that NF-κB signaling pathway was up-regulated (Fig. 7a). When QNZ (10 nM), an inhibitor of the NF-κB signaling pathway, or siRNA against *KFKB1* was added along with DOX starting at D4, flow cytometry analysis at D14 showed that the promotion of hematopoiesis (especially myelogenesis) and the impair of erythrogenesis by *HOXA9* overexpression were partially or completely abolished (Fig. 7b, Fig. S3). These observations indicated that the effects of *HOXA9* overexpression on hematopoiesis during the late stage were closely related to NF-κB signaling.

### The effects of *HOXA9* overexpression on hematopoiesis were related to a change in cell cycle status, which can be reversed by inhibition of NF-κB signaling

Cell cycle assays revealed a significant increase in the proportion of cells in S phase in co-cultures induced starting at D4, whereas addition of QNZ or siRNA against *NFKB1* at the same time abolished this increase. Similar effects were observed for all cells, as well as for the CD45+ population. This observation indicated that the increase in proliferation is the main cellular mechanisms responsible for the significant expansion of the CD45+ population, which is closely related to NF-κB signaling. When NF-κB signaling was blocked by a specific inhibitor or siRNA against *NFKB1*, all of these effects disappeared (Fig. 7c).

### Changes in gene expression underlie *HOXA9*-promoted hematopoiesis via up-regulation of NF-κB signaling and the cell cycle

**Fig. 8:**
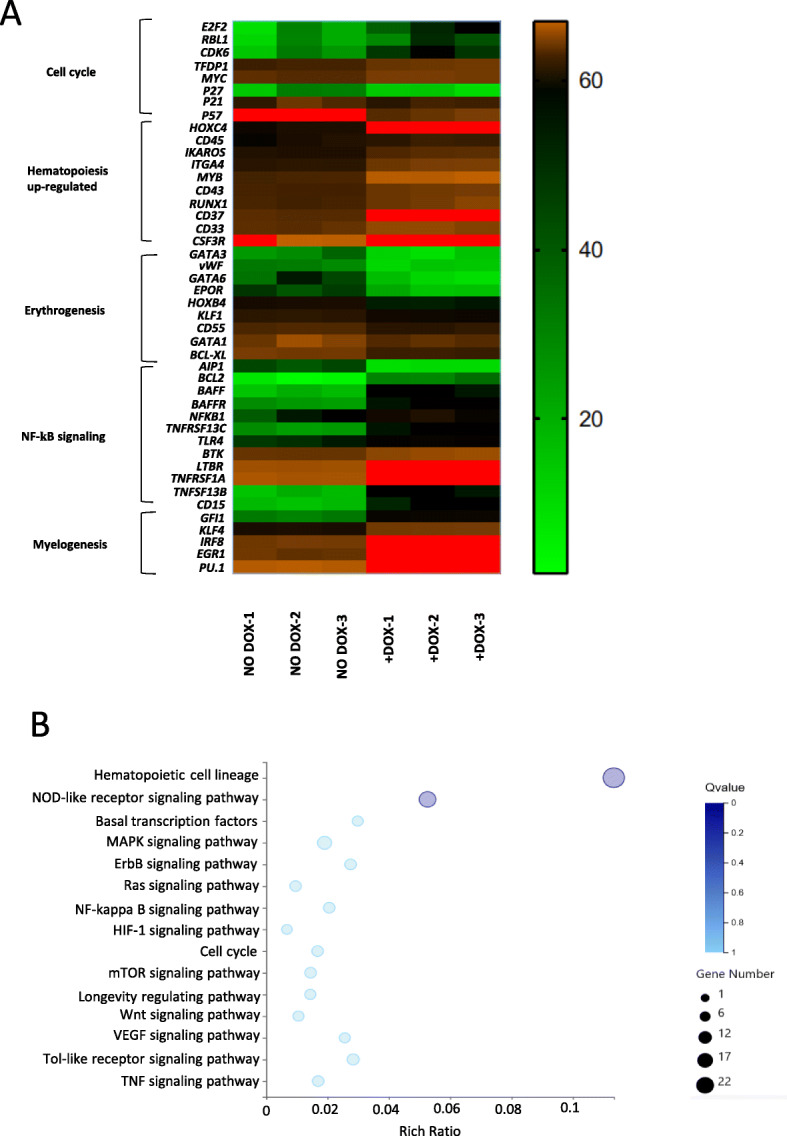
The bioinformation analysis of *HOXA9* overexpression. **a** Heatmaps of RNA-seq results of GFP+ cells sorted from D4-induced *HOXA9*/hESCs co-cultures or control cells without induction at D14. Pairwise comparison of transcription levels of selected genes. Heatmaps of selected genes associated with cell cycle, hematopoietic potential, and the NF-κB signaling pathway. In each row, red, black, and green reflect high, medium, and low expression, respectively. **b** The rich factor plot of KEGG pathway enrichment analysis of RNA-seq results for signaling pathway, in which item the degree of color stands for the *P* value and the size of node stands for the gene count

To elucidate the mechanism by which *HOXA9* promoted myeloid progenitors, GFP+ cells of corresponding induced co-cultures were subjected to gene analysis controlled by the non-induced co-cultures. The surface markers of hematopoiesis (Kennedy et al. 2012), such as CD43, CD45, CD37, CD33, and other genes that promote the development of myeloid progenitor cells and hematopoietic differentiation, such as *HOXC4*, *RUNX1*, and *MYB*, were up-regulated, whereas genes related to erythroblast development, such as *GATA1*, *EPOR*, and *GATA3*, were down-regulated. Genes that promote the G1/S phase transition, such as *E2F2*, *RBL1*, and *CDK6*, were up-regulated, and cyclin-dependent kinase inhibitors, such as *P21*, *P27, P57*, were down-regulated. Genes related to the NF-κB signaling pathway, such as *NFKB1, BAFF*, and *BAFFR*, were up-regulated. Genes related to myelogenesis, such as *PU.1, EGR1,* and *GFI1* were up-regulated. The rich factor plot of KEGG pathway enrichment analysis of RNA-seq results for signaling pathway also indicated the up-regulation of the NF-κB signaling pathway. These data indicate that *HOXA9* promoted hematopoiesis, and especially myelogenesis while impaired the erythrogenesis by up-regulating NF-κB signaling and the cell cycle (Fig. 8).

### *HOXA9* mRNA expression was up-regulated at D6 and gradually decreased at the late stage of hematopoietic differentiation in the AGM-S3 co-culture system

qRT-PCR analysis at various time points showed that *HOXA9* expression at the mRNA level was up-regulated at D6 dramatically and gradually decreased at the late stage of hematopoietic development for non-transgenic H1 hESCs in the AGM-S3 co-culture system: *HOXA9* had much lower expression during mesoderm induction (D0–D4) than the one during hematopoietic differentiation (D6–D14), and a small increase could be observed at D14 (Fig. S4).

## Discussion

*HOXA9* plays a crucial role in blood cell development. Defects in *HOXA9* lead to impaired hematopoiesis and abolish the ability of HSCs to repopulate irradiated recipients (Lawrence et al. 2005). Overexpression of *HOXA9* dramatically increases the efficiency of hematopoiesis (Ramos-Mejia et al. 2014; Ohno et al. 2013). However, the role of *HOXA9* during human hematopoiesis, and its potential to increase the production of hematopoietic cells and cause abnormal hematopoiesis, need to been further studied in detail with higher accuracy.

To address this problem, we used a novel *piggyBac* Tet-on system, developed previously by us, to detailly detect the effects of enforced expression of *HOXA9* during different co-culture stages of hematopoiesis with much higher accuracy. AGM-S3 in vitro co-culture system had also been used to reveal the function of *HOXA9* in hematopoietic differentiation. In such systems, induction of *HOXA9* at various times revealed distinctive, primarily positive, functions of *HOXA9* at different stages of mesodermal development and hematopoiesis, suggesting that the gene has the potential to improve human hematopoiesis.

Induction of *HOXA9* from D0 did not significantly influence production of KDR+ cells that marked mesoderm (Vodyanik et al. 2006) (Fig. 2a, Fig. S2A) while it caused that the CD34+ cells and derived populations had significantly decreased at D8 and D14 (Fig. 2b, Fig. S2B). KDR+ cells isolated from the D1 co-culture did not produce CD34+ cells upon DOX induction (Fig. 3a). These observations demonstrate that induction of *HOXA9* from the earliest stage (D0) can block the mesoderm–hemogenesis transition without influencing the induction of the mesoderm. This is similar to what has been observed before for other hematopoiesis-related genes, such as *RUNX1* (Chen et al. 2017), indicating that the overexpression of these genes at the earliest stage of co-culture might be harmful to the mesoderm–hemogenesis transition.

Induction of *HOXA9* from D4 or later increased the production of CD34 + CD43− (endothelial cells) (Chiang et al. 2018), CD34 + CD43+ (hematopoietic progenitors) (Vodyanik et al. 2006; Duan et al. 2018; Nakajima-Takagi et al. 2013), and CD34 − CD43+ populations at D8, as well hematopoietic stem/progenitor cells and myeloid progenitors, but not erythroid progenitors at D14 (Fig. 2b, c) (Vodyanik et al. 2006; Choi et al. 2009). Further culture of KDR+ cells isolated from D4 co-cultures after DOX induction produced more hematopoietic progenitor cells from these cells, especially CD34 − CD45+ cells. This indicated that KDR+ cells at a late stage of mesoderm induction could be induced to undergo hematopoietic differentiation by overexpression of *HOXA9* (Yang et al. 2008; Brumatti et al. 2013).

When *HOXA9* was induced from D4, the CD34 − CD45+ population dramatically increased at D14 by about 4 to 5-fold relative to the non-induced controls (Fig. 2c). qRT-PCR analysis of non-trangenic H1 hESCs co-cultured with AGM-S3 revealed that mRNA expression of *HOXA9* significantly increased at D6 and gradually dropped thereafter (Fig. S4). Therefore, overexpression of *HOXA9* from D4 dramatically promoted hematopoiesis is reasonable for D4 is very near to the windows of *HOXA9* function. This might explain why D4 is the key period during which *HOXA9* promotes hematopoiesis.

To elucidate the detailed mechanism by which *HOXA9* induction starting after D4 promotes hematopoiesis, we isolated the CD34 + CD43+ population by sorting *HOXA9*/hESCs co-cultured with AGM-S3 at D8 or D14, and then subjected them to further hematopoietic culture to test their potential for hematopoiesis (Vodyanik et al. 2011).

DOX induction during subsequent culture of the CD34 + CD43+ population isolated from the D8 co-culture dramatically increased the production of CD34 − CD43+ and CD34 − CD45+ populations, which overlapped with the CD43 + CD45+ population (Yang et al. 2008; Vodyanik et al. 2011). When DOX induction was performed from the starting day of suspension culture, the production of CD34 − CD43+ and CD34 − CD45+ populations, was significantly higher than in the absence of DOX induction (Fig. 4). It is clear that induction of *HOXA9* from D8 promoted their hematopoietic potential, as revealed by the increase in the production of CD34 + CD43+, CD34 − CD43+, CD34 + CD45+, CD34 − CD45+ cells detected at D14 (Fig. 2c).

CD34 + CD43+ population isolated from D14 co-cultures was further cultured in myeloid expansion medium, which yielded a population consisting almost entirely of 100% CD34 − CD45+ cells after DOX induction. Induction of *HOXA9* from starting day of suspension culture produced much greater CD34 − CD45+ cells than 53.4%, the value without any induction (Fig. 5). The colony culture assay also revealed that GFP+ cells, from either whole cocultures or the CD34 + CD43+ population that overexpress *HOXA9*, can produce a higher number of CFU-GM colonies than their non-induced counterparts (Fig. 6A). On the contrary, if these cells were further cultured in erythroid expansion medium, it only yielded much less GPA + CD71+ populations (0.2%) after DOX induction compared with non-induced control cells (5.4%) (Fig. 5). The corresponding colony culture assay also showed that the number of CFU-E colonies derived from the induced co-culture cells was much lower than their non-induced counterparts, which were not caused by the overexpression of *GFP* (Fig. 6A). Taken together, above results indicated the overexpression of *HOXA9* (not *GFP*) from D4 lead to a significant increase in the myelogenic potential of the CD34 + CD43+ population isolated from D14 co-cultures while a decrease in their erythrogenic potential. To elucidate the molecular mechanism of *HOXA9* in hematopoiesis, we investigated the relevant signaling pathways. The function of *HOXA9* is closely relative to NF-κB signaling (Trivedi et al. 2008; Han et al. 2019). The research of Menendez’s group also speculated that NF-κB signaling might be a potential candidate to participate in the promotion effects of *HOXA9* on human hematopoiesis according bioinformation analysis (Ramos-Mejia et al. 2014) while no further research was reported, so we focused on this signaling and explored its relationship to *HOXA9*. The results showed that NF-κB signaling was significantly up-regulated when *HOXA9* was induced from D4 (Fig. 7a). QNZ, an inhibitor of NF-κB signaling, efficiently blocked the promotion of myelogenesis and impairment of erythrogenesis caused by *HOXA9* overexpression when it was added along with DOX starting at D4. The siRNA against *NFKB1* had similar effects with QNZ (Fig. 7b). These observations indicated that NF-κB signaling was highly relevant to these effects, consistent with a hypothesis proposed in a previous study (Trivedi et al. 2008; Chen et al. 2007; Zhong et al. 2018; Trivedi et al. 2007; Ledoux and Perkins 2014; Yu et al. 2016; Yin et al. n.d.; Panepucci et al. 2010; Kuo et al. 2013). In some reports, continued expression of *HOXA9* was shown to inhibit the endothelial cell (EC) activation pathway by interfering with the transcriptional activity of NF-κB (Trivedi et al. 2008; Trivedi et al. 2007), while in our co-culture system *HOXA9* induction from D4 or later increased NF-κB transcriptional activity, which might involve a different molecular mechanism.

Cell cycle regulation is extremely important for hematopoiesis, which is always associated with up-regulation of the cell cycle (Hao et al. 2016; Palis 2016) . Cell cycle analysis at D14 revealed that, after induction of *HOXA9* from D4, a greater proportion of GFP+ cells, including GFP + CD45+ cells, were in S-phase, and that this was inhibited by QNZ or siRNA against *NFKB1* (Fig. 7c). This observation suggests that *HOXA9* might be able to alter cell cycle status, with the participation of NF-κB signaling (Panepucci et al. 2010; Kuo et al. 2013; Poulos et al. 2016). This was further confirmed by the corresponding heatmaps and the rich factor plot of KEGG pathway enrichment analysis of of total RNA Sequencing data (Fig. 8).

## Conclusions

In this study, we analyzed the molecular/cellular mechanism of *HOXA9* involvement in blood physiology using a unique AGM-S3 co-culture system and a Tet-on inducible system based on *piggy*Bac. The results indicated that induction of *HOXA9* from D4 or later to D14 during co-culture with AGM-S3 efficiently promoted hematopoiesis and myelogenesis while obviously impaired erythrogenesis, probably via changes in NF-κB signaling and cell cycle status. The detailed molecular/cellular mechanism of *HOXA9* in hematopoietic differentiation should be further explored in the future.

## Materials and methods

### Establishment of *HOXA9* inducible transgenic hESC lines

The coding region of *HOXA9* was inserted between the *Swa*I and *Eco*RI sites of PB-Tet-on-OE to construct PB-Tet-on-GFP-T2A-hHOXA9, which was co-transfected in H1 hESCs along with the helper vector PB200PA-1 using Lipofectamine 3000 (Invitrogen). Positive colonies were selected with 1 μg/ml puromycin, and then passaged using ReleSR (STEM CELL Technologies) to establish an inducible hESC line (*HOXA9*/hESC) that co-expressed GFP. Induction by DOX was confirmed by quantitative reverse-transcription PCR (qRT-PCR) and western blot analyses, and pluripotency was confirmed by western blot analyses of SOX2, OCT4, and NANOG. The method is described in detail in Supplementary Materials and qPCR primer pairs (HOXA9–2 and GAPDH-2) were listed in Table S1.

### Co-culture of hESCs with AGM-S3 cells

To perform hematopoietic differentiation, the H1 hESC line (generously provided by Prof. Tao Cheng) was co-cultured with AGM-S3 (a mouse stroma-derived cell line) as reported previously (Mao et al. 2016; Xu et al. 1998). This study was approved by the institutional ethics committee of the Institute of Blood Transfusion, Chinese Academy of Medical Sciences and Peking Union Medical College (CAMS & PUMC). Briefly, undifferentiated H1 hESCs were seeded on irradiated AGM-S3 cells, cultured in hPSC maintenance medium (Dulbecco’s modified Eagle’s medium (DMEM) with high glucose, F-12 nutrient mixture, 20% knockout serum replacement (KSR; Gibco), 1% L-glutamine, 1% non-essential amino acid solution (NEAA; Gibco), and 5 ng/mL basic FGF (b-FGF; Wako)) for 3 days, and then switched to hematopoiesis-inducing medium (Iscove’s modified Dulbecco’s medium (IMDM) containing 10% fetal bovine serum (FBS; Hyclone), 1% NEAA (Gibco), 60 ng/mL ascorbic acid (Sigma), and 20 ng/mL vascular endothelial growth factor (VEGF; Wako)) and referred to as Day 0 [D0]. The co-cultured cells were dissociated with 0.05–0.25% trypsin/EDTA (ethylenediaminetetraacetic acid) solution (Invitrogen) at the indicated times after D0 and then subjected to flow cytometry or other manipulations.

### Flow cytometry and cell sorting

Co-cultured cells were dissociated with 0.25% trypsin-EDTA solution (Invitrogen), filtered through a 40 μm nylon mesh to obtain a single-cell suspension, stained with corresponding antibodies (Table S2), and subjected to flow cytometry analysis using a FACSCanto II system, or cell sorting using a FACSJazz™ Sorter (BD Biosciences). All flow cytometry data were analyzed using FlowJo 10. The corresponding populations were isolated at different stages and performed the consequent hematopoietic culture as Fig. S1 presented.

### Hematopoietic differentiation assay using full-lineage hematopoietic differentiation medium

Non-induced *HOXA9*/hESC co-cultures at D8 were dissociated by treatment with 0.25% trypsin solution and stained with 7-AAD and the combination of anti-CD34/CD43 antibodies. CD34 + CD43+ cells were sorted from the corresponding co-cultured cells, and about 5 × 10^3^ sorted cells were resuspended in 250 μl full-lineage hematopoietic differentiation (FLHD) medium (IMDM containing 10% FBS, 100 ng/mL SCF, 100 ng/mL IL-6, 10 ng/mL IL-3, 10 ng/mL FL, 10 ng/mL TPO, and 4 IU/mL EPO) for 12 days treated with or without DOX, changed media every day and finally performed flow cytometry analysis.

### Differentiation of myeloid and erythroid with suspension culture

Non-induced *HOXA9*/hESC co-cultures at D14 were dissociated by treatment with 0.25% trypsin solution and stained with 7-AAD and the combination of anti-CD34/CD43 antibodies. CD34 + CD43+ cells were sorted from the corresponding co-cultured cells, and about 5 × 10^3^ sorted cells of each type were resuspended in 250 μl myeloid expansion medium (Cat# 02693; STEMCELL Technologies) or erythroid expansion medium (StemSpan™ SFEM II (Catalog#09605, STEMCELL Technologies) containing 10^− 6^ M Dexamethasone, 4 U/ml EPO, 100 ng/ml IL-6, and 100 ng/ml SCF), then seeded into 48-well plates to be further cultured with or without DOX induction for 8 days, with a medium exchange every other day. At the end of 8 days, the cells were subjected to flow cytometry.

### Colony culture assay

GFP/hESC line can inducible express only GFP without HOXA9 protein, which was established before using PB-Tet-on-OE (Chen et al. 2017). The non-induced *HOXA9*/hESC co-culture cells and the GFP+ cell fractions of *HOXA9*/hESC or GFP/hESC co-culture cells induced from D4, or the CD34 + CD43+ population in these cells were sorted by flow cytometry. The hematopoietic potentials of these co-culture cells were assessed by culture on methylcellulose (Cat# H4320; STEMCELL Technologies) supplemented with 100 ng/ml stem cell factor (SCF), 100 ng/ml interleukin-6 (IL-6), 10 ng/ml interleukin-3 (IL-3), 10 ng/ml Fms-related tyrosine kinase 3 ligand (FL), 10 ng/ml thrombopoietin (TPO), 10 ng/ml granulocyte-macrophage colony-stimulating factor (GM-CSF), 4 units/ml erythropoietin (EPO), and 1% penicillin/streptomycin, and then incubated in 5% CO_2_ at 37 °C for 14 days. At 7–10 days the colony number of colony-forming unit–erythroid (CFU-E) was calculated. At 14 days the colony numbers of burst-forming unit-erythroid (BFU-E), colony-forming unit–mixed (CFU-Mix), and colony-forming unit–granulocyte/macrophage (CFU-GM) were calculated.

### Testing the activation of NF-κB signaling and detecting the antagonistic effects of NF-κB signaling inhibition on *HOXA9* overexpression at the late stage of hematopoiesis

D4-induced *HOXA9*/hESCs co-cultured with AGM-S3 cells were subjected to qRT-PCR detection at D14 using *NFKB1* and *NFKB2* primer pairs. Order QNZ (an inhibitor of the NF-κB signaling pathway) from Selleck inc. and dissolve it using DMSO. Order siRNAs against *NFKB1* from Sangon Biotech Inc. D4-induced *HOXA9*/hESCs co-cultured with AGM-S3 cells were treated from D4 with 10 nM QNZ (Selleck) or the equal volume of DMSO as control, or treated from D4 with 20 nM siRNA against *NFKB1* or the equal concentration of control siRNA, and then subjected to qRT-PCR, flow cytometry, and cell cycle analysis at D14. Untreated co-culture cells were used as a negative control. The method is described in detail in Supplementary Materials and qPCR primer pairs were listed in Table S1.

### RNA-Seq

Non-induced or D4-induced *HOXA9*/hESC co-cultures at D14 were dissociated by treatment with 0.25% trypsin solution. Non-induced co-culture cells and GFP+ fractions of D4-induced co-culture cells were sorted, and about 1 × 10^6^ cells were treated with 1 ml TRIzol (Life Technologies). RNA was extracted and isolated, and quantified. Total RNA Sequencing was performed by BGI Tech. Analysis of gene functions was performed using the Dr. Tom Multi-Group Data Mining System (http://report.bgi.com). The experiments were repeated three times.

### Detection of *HOXA9* mRNA expression during co-culture of non-transgenic H1 hESCs with AGM-S3

To explore the molecular mechanism of *HOXA9* in hematopoietic development, *HOXA9* mRNA levels in co-culture cells of non-transgenic H1 hESC were monitored by qRT-PCR every other day, including on D0, D2, D4, D6, D8, D10, D12, and D14. The method is described in detail in Supplementary Materials and qPCR primer pairs (HOXA9–1 and GAPDH-1) were listed in Table S1.

### Statistical analysis

All data are presented as means ± SD; statistical analyses were performed using Student’s t test. *P* < 0.05 was considered significant.

## Supplementary Information

**Additional file 1: Supplementary Materials.** Supplementary material is available at *Cell Regeneration* online.

## Data Availability

Data have been deposited at https://submit.ncbi.nlm.nih.gov/subs/biosample/ under the number SAMN 12721158.

## Abbreviations

AGM: Aorta/gonad/mesonephros
BFU-E: Burst-forming unit-erythroid
b-FGF: Basic FGF
CDS: Coding sequence
CFU-E: colony-forming unit-erythroid
CFU-GM: Colony-forming unit-granulocyte/macrophage
CKI: Cyclin-dependent kinase inhibitor
CMV: Cytomegalovirus
CFU-Mix: Colony-forming unit-mixed
DOX: Doxycycline
DMEM: Dulbecco’s modified Eagle’s medium
EPO: Erythropoietin
FBS: Fetal bovine serum
FL: Fms-related Tyrosine Kinase 3 Ligand
FLHD: Full-lineage hematopoietic differentiation
GFP: Green fluorescent protein
GM-CSF: Granulocyte-macrophage colony-stimulating factor
hESC: Human embryonic stem cells
hiPSC: Induced pluripotent stem cells
IL-3: Interleukin-3
IL-6: Interleukin-6
IMDM: Iscove’s modified Dulbecco’s medium
KSR: Knockout serum replacement
NEAA: Non-essential amino acid
qRT-PCR: Quantitative reverse transcription PCR
*RUNX1*: Runt-related transcription factor 1
SCF: Stem cell factor
TPO: Thrombopoietin
VEGF: Vascular endothelial growth factor
