# Supplementary material for: Overexpression of HOXA9 upregulates NF-κB signaling to promote human hematopoiesis and alter the hematopoietic differentiation potentials
Source: Cell Regen. 2021 Jan 11;10:9. doi: 10.1186/s13619-020-00066-0 (PMC7797385; doi:10.1186/s13619-020-00066-0)
Supplement: Supplementary file 1 — Additional file 1: Supplementary Materials. Supplementary material is available at Cell Regeneration online. [file 13619_2020_66_MOESM1_ESM.pdf]

Supplemental Information

Supplemental materials and methods

Cell cycle analysis

At D14 non-induced *HOXA9*/hESC co-cultures or D4-induced *HOXA9*/hESC co-cultures with or without 10 nM QNZ or 20 nM siRNA against *NFKB1* were treated with 10 μM BrdU for 12 h, dissociated by treatment with 0.25% trypsin solution, stained with anti-CD45 antibody, and then subjected to analysis of cell cycle status using the APC-BrdU Flow Kit (BD Biosciences), which was visualized by flow cytometry analysis.

May-Grunwald-Giemsa staining

Cells from BFU-E colonies were harvested and spun onto glass slides using the Cell Cytospin 4 (Thermo Fisher Scientific), dyed in May-Grunwald solution (MERCK No. 101424) for 10 min, and further stained in Giemsa B solution for 2–3 min. After washing, Giemsa A solution (MERCK No. 10924) and Giemsa B solution were added at a 1:20 ratio for 20 min, and the sample was washed with water and air dried. Samples were imaged under an oil emersion lens on an Olympus BX53 microscope.

Quantitative reverse transcription PCR (qRT-PCR)

Total RNA was extracted from 5×10^5 cells using 500 µl TRIzol (Life Technologies). Complementary DNA was synthesized using a reverse transcription kit (Bio-Rad). Each 20 µl reaction contained 4 µl of 5× Mixture, 1 µl reverse transcriptase, 1 µg of total RNA, and nuclease-free water to 20 µl. The program used for reverse transcription was as follows: 25°C for 5 min, 42°C for 30 min, 85°C for 5 min, and hold at 4°C. qPCR was performed using the Fast Start Universal SYBR Green Master (Roche) on a CFX96™ real-time system (Bio-Rad). Each 15 µl reaction contained 7.5 µl of 2× Mixture, 0.4 µl of each primer (10 µM), 4.7 µl H_2O, and 2 µl complementary DNA (cDNA). The conditions were as follows: denaturation for 10 min at 95°C, followed by 45 cycles of 95°C for 15 sec, 58°C for 30 sec, and 72°C for 30 sec. Glyceraldehyde 3-phosphate dehydrogenase (*GAPDH*) served as an internal control. The primers for qRT-PCR were listed in Table S1. Among them the primer pairs of HOXA9-1 and GAPDH-1 were specially designed for human *HOXA9* and *GAPDH* transcripts, and cannot be used to detect the transcripts of corresponding mouse genes. They were used to perform the qRT-PCR detection of endogenous human *HOXA9* of un-modified H1 hESC co-cultures during hematopoiesis so as to exclude the interference of AGM-S3 cells, which results are presented in Figure S4. For primer pair of HOXA9-1 was designed in un-translated region (UTR) of human *HOXA9* transcripts it couldn’t detect the transcript of exogenously expressed

HOXA9. The primer pairs of HOXA9-2 and GAPDH-2 designed according to the coding region sequences of human *HOXA9* and *GAPDH* transcripts and mouse *Hoxa9* and *Gapdh* transcripts were used to perform the qRT-PCR detection of exogenously expressed human *HOXA9* of induced or non-induced *HOXA9*/hESCs cultured in hESC medium (mTeSR1, Stem cell, USA), which results were not interfered by the AGM-S3 cells, and presented in Figure 1C.

West blot analysis

Cells were lysed in RIPA buffer (Sigma) with shaking on ice for 30 min. After centrifugation (12,000 x g, 4℃, 5 min), the protein concentration in the extract was assayed using the classical BCA method. Proteins were denatured at 100°C for 5 min and then electrophoresed by denaturing SDS-PAGE (5% separation gel/10% stacking gel) according to the standard protocol of the Bio-Rad Mini-PROTEAN Tetra Cell electrophoresis system. Proteins were transferred to a PVDF membrane using a Bio-Rad Trans-Blot cell in 1× TBST (10 mM Tris-HCl, pH 8.0, 150 mM NaCl, and 0.05% Tween 20). Thereafter, membranes were incubated with anti-SOX2 (diluted 1:1000; Cat. # sc-365823, SANTA CRUZ), anti-OCT3/4 (diluted 1:1000; Cat. # sc-5279, SANTA CRUZ), anti-NANOG (diluted 1:1000; Cat. # sc-293121, SANTA CRUZ), anti-HOXA9 (diluted 1:1000; Cat. # ab140631, Abcam), or anti-GAPDH (diluted 1:1000; Cat. # KC-5G4, KANG CHEN) mouse monoclonal IgGs in blocking solution (1× TBST containing 5% non-fat milk powder) overnight at 4°C. Membranes were then washed with 1× TBST, incubated with horseradish peroxidase-conjugated goat anti-mouse IgG H&L (diluted 1:1000; Cat. # ZB-2305,

ZSBIO) or Donkey Anti-rabbit IgG (diluted 1:1000; Cat. # bs-0295D, Bioss) in blocking solution, washed again with 1× TBST, and finally developed using an Amersham ECL Prime western blotting detection kit (Cat. # RPN2232, GE Healthcare). Signals were quantified using an ImageQuant LAS4000 mini system. Molecular weights were calculated by reference to the pre-stained protein ladder (Cat. # 26626, PageRuler).

Supplemental Figure Legends

Supplemental Figure 1. After determination of the best mode of *HOXA9* induction (induction from D4 or later), the following cultures were initiated: KDR+ cells sorted at D1 or D4 from non-induced *HOXA9*/hESC co-cultures were re-plated on AGM-S3 with or without DOX induction for 10 or 12 days; CD34+CD43+ cells sorted from non-induced co-cultures at D8 were re-plated on AGM-S3 with or without DOX induction for 10 days; CD34+CD43+ cells sorted from non-induced co-cultures at D14 were cultured in myeloid or erythroid expansion medium with or without DOX induction for 8 days. The results are shown in Figures 3–5.

Supplemental Figure 2. Co-cultured *HOXA9*/hESCs were treated with DOX from D0, D2, D4, D6, D8, D10, or D12, and analyzed by flow cytometry using 7-AAD and the combination of the antibodies against **(A)** KDR (at D4), **(B)** CD34/CD43 (at D8), or (C) CD34/CD43, CD34/CD45, and GPA/CD71 (at D14). Non-induced co-cultures and the GFP+ fraction

of co-cultures treated with DOX were compared using flow cytometry analysis. Overexpression of *HOXA9* from D4 caused the greatest stimulation of hematopoiesis and myelogenesis, but impaired the erythrogenesis.

Supplemental Figure 3. The D14 *HOXA9*/hESCs co-cultures treated with or without DOX or treated with both DOX and 10 nM QNZ/ 20 nM siRNA against NFKB1 from D4 were subjected to flow cytometry analysis at D14.

Supplemental Figure 4. Un-modified H1 hESCs co-cultured with AGM-S3 were collected at D2, D4, D6, D8, D10, D12, or D14, and *HOXA9* mRNA expression was detected by qRT-PCR analysis using primer pairs of HOXA9-1 and GAPDH-1. *GAPDH* was used as an internal control.

TABLE S1
Primers used for qRT-PCR analysis

Gene	Forward primer (5′→3′)	Reverse primer (5′→3′)
NF-KB1	GAAGAGGAAGAAAATGGTGGAGT	AAACACAGAGGCTGGTTTTGTAA
NF-KB2	GAGAGAAGCCGCAACCAGAG	GGGTTGTAGCAACTCTCCATGTC
HOXA9-1	CGAGTGATGCCATTTGGGCTTATTTAG	AGGGTGGGGGTGAGAGAAGG
HOXA9-2	GCGCCTTCTCTGAAAACAAT	CAGTTCCAGGGTCTGGTGTT
GAPDH-1	CGACAGTCAGCCGCATCTTCTT	TTCCCCATGGTGTCTGAGCG
GAPDH-2	GTCTCCTCTGACTTCAACAGCG	ACCACCCTGTTGCTGTAGCCAA

TABLE S2
Flow cytometry antibodies

Antibodies	Brand	Antibody channels	Cat
CD309(KDR)	BD	PE	560494
CD34	BD	APC	555824
CD43	BD	PE	560199
CD45	BD	PE-CY7	557748
CD235a(GPA)	BD	APC	551336
CD71	BD	PE	561938
7-AAD	BD	7-AAD	559925

Figure S1

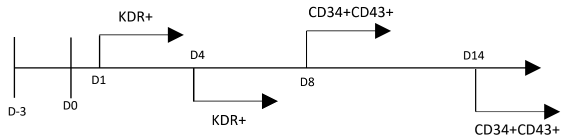

Figure S2

A Flow cytometry analysis at D4

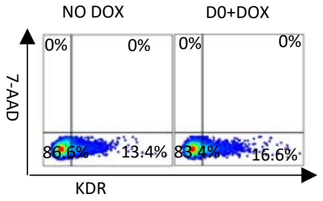

B Flow cytometry analysis at D8

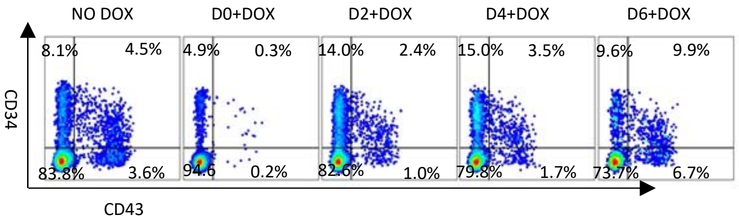

C Flow cytometry analysis at D14

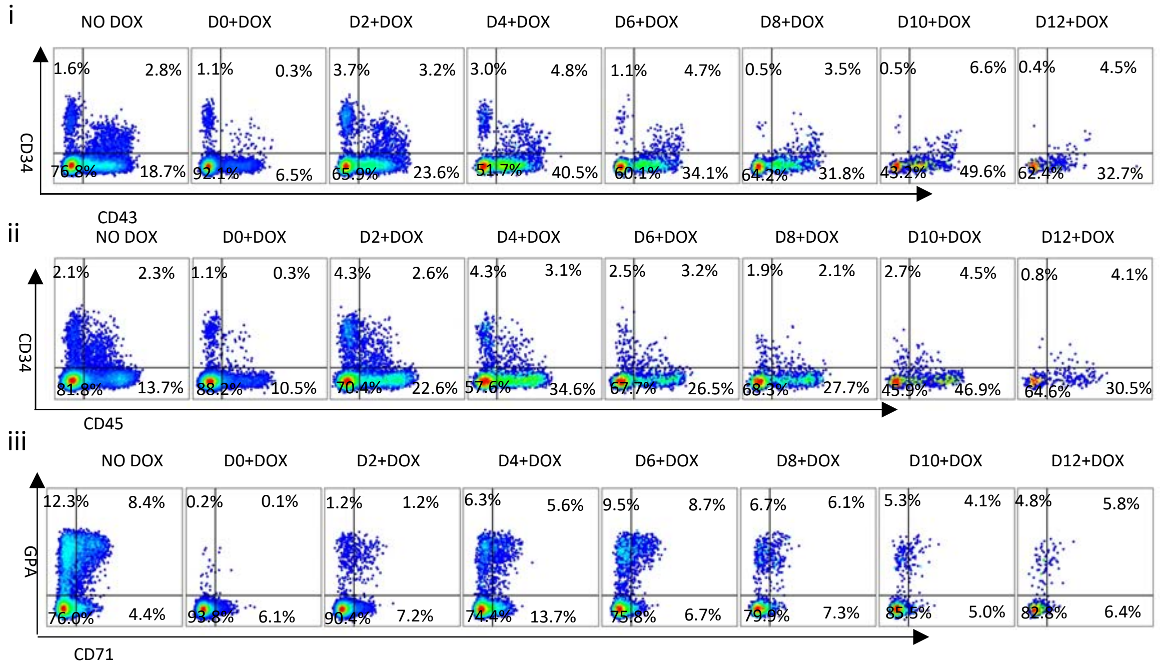

D Flow cytometry FMO at D14

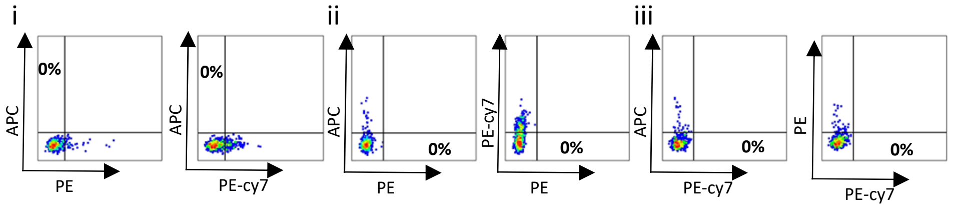

Figure S3

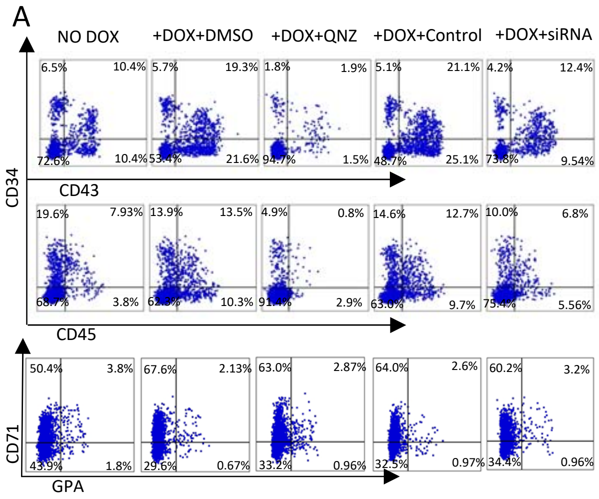
A
NO DOX
+DOX+DMSO
+DOX+QNZ
+DOX+Control
+DOX+siRNA
CD34
CD43
CD45
CD71
GPA

Figure S4

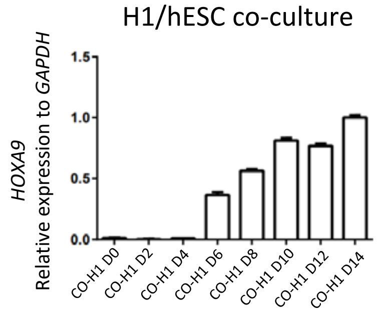
H1/hESC co-culture
HOXA9
Relative expression to GAPDH
1.5
1.0
0.5
0.0
CO-H1 D0
CO-H1 D2
CO-H1 D4
CO-H1 D6
CO-H1 D8
CO-H1 D10
CO-H1 D12
CO-H1 D14
